# A single chromosome strain of *S. cerevisiae* exhibits diminished ethanol metabolism and tolerance

**DOI:** 10.1186/s12864-021-07947-x

**Published:** 2021-09-22

**Authors:** Tyler W. Doughty, Rosemary Yu, Lucy Fang-I Chao, Zhongjun Qin, Verena Siewers, Jens Nielsen

**Affiliations:** 1 Department of Biology and Biological Engineering, Chalmers University of Technology, SE-412 96 Gothenburg, Sweden; 2 Novo Nordisk Foundation Center for Biosustainability, Chalmers University of Technology, SE-412 96 Gothenburg, Sweden; 3 Key Laboratory of Synthetic Biology, CAS Center for Excellence in Molecular Plant Sciences, Shanghai Institute of Plant Physiology and Ecology, Chinese Academy of Sciences, Shanghai, China; 4 Novo Nordisk Foundation Center for Biosustainability, Technical University of Denmark, DK-2800 Kongens Lyngby, Denmark; 5 BioInnovation Institute, DK-2200 Copenhagen N, Denmark

## Abstract

**Background:**

Eukaryotic organisms, like the model yeast *S. cerevisiae*, have linear chromosomes that facilitate organization and protection of nuclear DNA. A recent work described a stepwise break/repair method that enabled fusion of the 16 chromosomes of *S. cerevisiae* into a single large chromosome. Construction of this strain resulted in the removal of 30 of 32 telomeres, over 300 kb of subtelomeric DNA, and 107 subtelomeric ORFs. Despite these changes, characterization of the single chromosome strain uncovered modest phenotypes compared to a reference strain.

**Results:**

This study further characterized the single chromosome strain and found that it exhibited a longer lag phase, increased doubling time, and lower final biomass concentration compared with a reference strain when grown on YPD. These phenotypes were amplified when ethanol was added to the medium or used as the sole carbon source. RNAseq analysis showed poor induction of genes involved in diauxic shift, ethanol metabolism, and fatty-acid ß-oxidation during growth on ethanol compared to the reference strain. Enzyme-constrained metabolic modeling identified decreased flux through the enzymes that are encoded by these poorly induced genes as a likely cause of diminished biomass accumulation. The diminished growth on ethanol for the single chromosome strain was rescued by nicotinamide, an inhibitor of sirtuin family deacetylases, which have been shown to silence gene expression in heterochromatic regions.

**Conclusions:**

Our results indicate that sirtuin-mediated silencing in the single chromosome strain interferes with growth on non-fermentable carbon sources. We propose that the removal of subtelomeric DNA that would otherwise be bound by sirtuins leads to silencing at other loci in the single chromosome strain. Further, we hypothesize that the poorly induced genes in the single chromosome strain during ethanol growth could be silenced by sirtuins in wildtype *S. cerevisiae* during growth on glucose.

**Supplementary Information:**

The online version contains supplementary material available at 10.1186/s12864-021-07947-x.

## Introduction

The nuclear genetic code of eukaryotic organisms is arranged as linear chromosomes that facilitate organization and protection of DNA [1–4]. Although chromosomes were observed as early as 1842, the characterization of the sequence and function of centromeres, telomeres, and autonomous replication sequences occurred much later [4, 5], as technology like DNA sequencing became more readily available [6]. Additional progress arose from disruption of chromosomal substructures, followed by characterization of mutants, and forward engineering. Examples of this paradigm include the sequencing, characterization, and forward engineering of mitotically segregated plasmids [2], as well as the design of artificial chromosomes in yeast (YACs) [7, 8]. Recent advances in high throughput sequencing have enabled sequencing and assembly of whole genomes, analysis of transcriptomes, and reconstruction of 3D chromosome structure [9–11]. The emergence of these tools coincides with advances in CRISPR-Cas9 genome editing [12], which has enabled scientists to create novel genomic structures.

Two recent reports applied the aforementioned technologies to create and analyze *S. cerevisiae* strains with one [13] or two [14] chromosomes via successive breaks of chromosome ends followed by repair/fusion [13, 14]. These end-to-end fusions are similar to events that can occur naturally during vast evolutionary timescales [15, 16], and enabled novel analyses of meiosis [14] and chromosomal folding [13] in the context of a few large chromosomes versus 16 smaller chromosomes. Intriguingly, the single chromosome strain from Shao et al. 2018 exhibited similar glucose phase growth rates and gene expression compared to the wildtype, despite significant changes in 3D chromosomal organization and interchromosomal interactions [13]. Additional comparative studies of reference and chromosome fusion strains to determine their phenotypic differences may shed light on the causes and consequences of chromosome organization, which will inform evolutionary biology and enable the design of synthetic chromosomes. In addition, analysis of these yeasts may shed light on previously uncharacterized mechanisms that involve multiple loci and are thus hard to detect with conventional synthetic biology techniques.

In this work, we investigated growth, gene expression, and metabolism of the single chromosome yeast strain from Shao et al. 2018 [13] compared with a reference strain during glucose and ethanol phase growth. We observed decreased biomass accumulation, decreased viability in the ethanol phase, and a dose dependent sensitivity to ethanol compared with the reference strain. Transcriptomics and metabolic modeling suggest that these phenotypes were influenced by improper activation of non-fermentable carbon source utilization and diauxic shift genes. Ethanol phase growth was rescued by nicotinamide, a sirtuin inhibitor. We hypothesize that the gene expression regime change from glucose to non-fermentable carbon sources is influenced by sirtuin de-repression, which is disrupted in the single chromosome strain.

## Results

### Profiling single chromosome strain growth

**Fig. 1:**
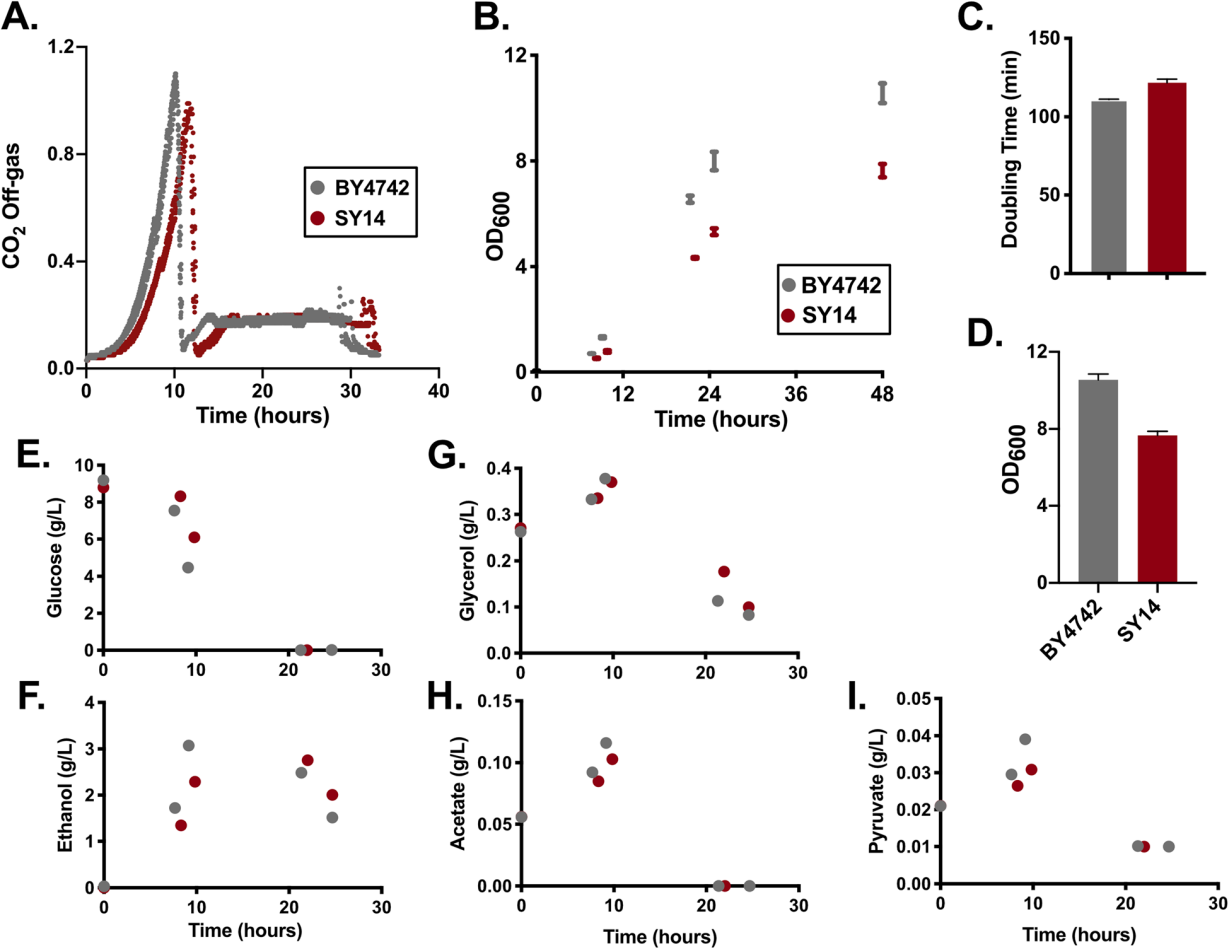
Batch cultivation of a single chromosome yeast strain. Reference (BY4742) and single chromosome (SY14) strains were analyzed via batch fermentation to monitor CO_2_ evolution (**a**) and biomass accumulation (**b**). Maximum glucose-specific doubling time (**c**) and the final OD_600_ at 48 h (**d**) were measured. **e**-**i** HPLC was used to monitor media composition at various timepoints

To characterize perturbations in growth and/or metabolism, we performed batch fermentations with triplicate bioreactors for the reference strain (*S. cerevisiae* strain BY4742) or the chromosomal fusion strain SY14, which has a single large chromosome instead of 16 distinct chromosomes (Fig. 1). Analysis of the CO_2_ evolution rate of the gas emitted from fermenters suggested that SY14 had an increased lag time prior to exponential growth on glucose (Fig. 1a). In addition, the doubling time during growth on glucose was increased by 8% for cultures of SY14 (120 min) compared with BY4742 (111 min) (Fig. 1c). Biomass accumulation, monitored via OD_600_, was diminished and became more apparent in the later stages of growth, culminating in 28% less biomass after 48 h of growth (Fig. 1b, d). Shake flask experiments showed a similar decrease in final biomass after 10 days (Additional File 1: Fig. S1). Despite a longer lag phase, SY14 cultures exhibited similar profiles of carbon source uptake, including complete uptake of glucose and production, followed by consumption of ethanol (Fig. 1e, f, g, h, i). Together, these findings indicate that SY14 exhibited a delay in growth after inoculation, had decreased glucose phase growth, and accumulated less biomass than the reference strain.

### The single chromosome strain (SY14) exhibits impaired growth on non-fermentable carbon sources and is sensitive to ethanol

**Fig. 2:**
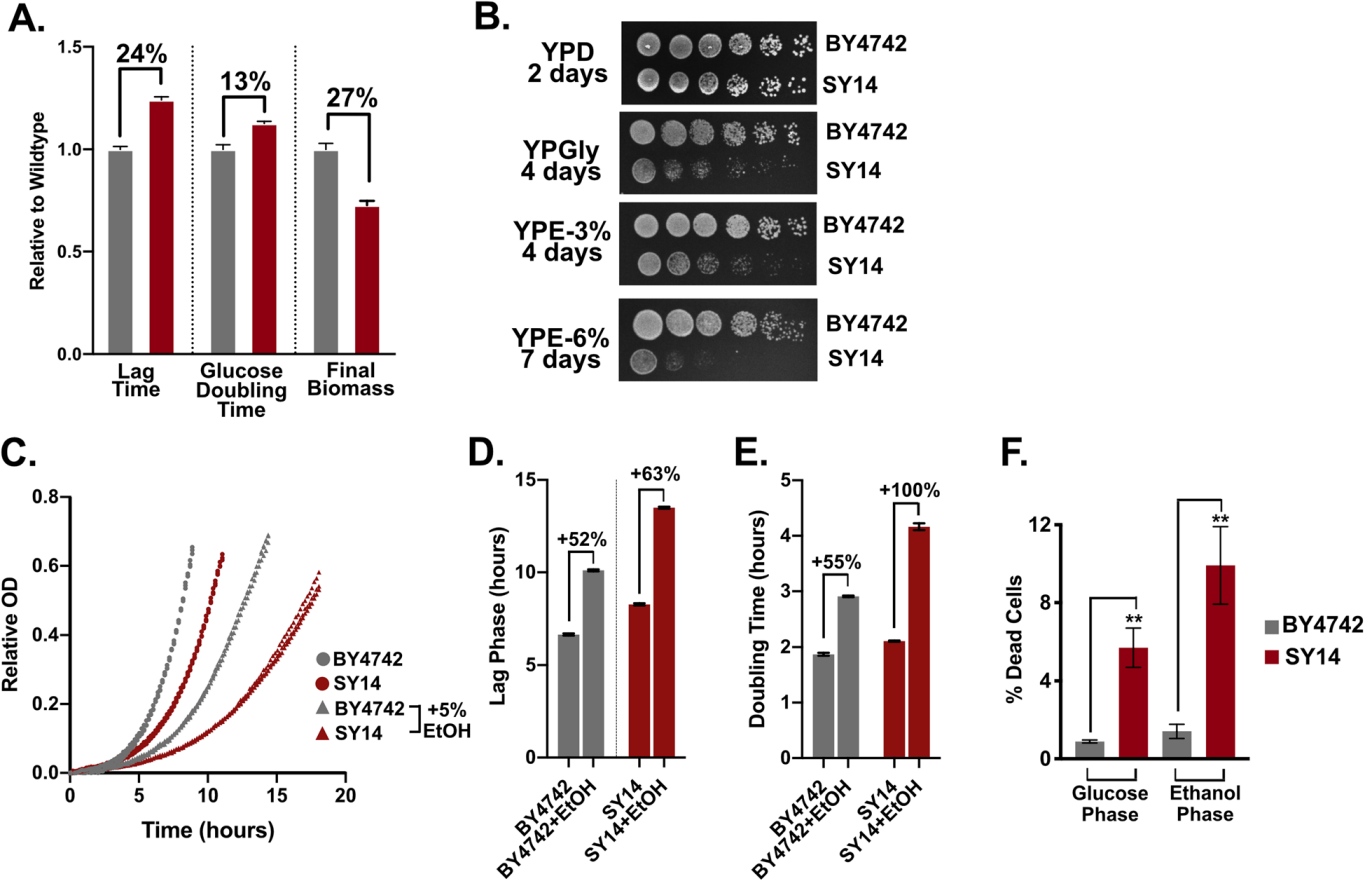
The single chromosome yeast grows slowly on non-fermentable carbon sources and is sensitive to ethanol. **a** Single chromosome and reference strain growth was monitored to determine the time to reach an OD_600_ of 0.25 (lag phase), maximum glucose growth, and final biomass at 48 h. **b** Growth on YP plates with varying carbon sources. **c** Glucose phase growth in YPD +/− 5% ethanol. Three replicates of each strain and condition were used to determine lag phase (**d**) and doubling time (**e**). **f** Reference (BY4742) and single chromosome (SY14) strains were analyzed for cell death using propidium iodide staining

The results presented in Fig. 1 warranted further analysis of the various stages of growth for SY14 and the reference strain. Similar to the fermentation results, microplate growth assays showed that cultures of SY14 exhibited a longer lag phase, increased doubling time during growth on glucose, and lower final biomass yield than BY4742 (Fig. 2a). Notably, the magnitude of these differences was larger for lag phase and final biomass than for glucose doubling time in microplate assays and fermenters. These differences suggested that SY14 cultures might struggle to grow on, and emerge from growth on non-fermentable carbon sources. To test this, we plated strains on glucose (YPD), ethanol (YPE), and glycerol (YPGly) plates (Fig. 2b). The results showed that growth of SY14 was diminished compared to BY4742 on non-fermentable carbon sources, but was similar on glucose. These phenotypes did not appear to be due to oxidative stress that might occur during growth on non-fermentable carbon sources, as addition of 3 mM H_2_O_2_ did not disproportionately influence SY14 doubling time or lag phase duration (Additional File 1: Fig. S2). Further, total protein levels (Additional File 1: Fig. S3a), as well as ribosomal RNA expression and processing were similar in wildtype and SY14 strains (Additional File 1: Fig. S3b).

The results in Fig. 2b showed that growth for SY14 was particularly diminished in the presence of 6% ethanol. To test for ethanol sensitivity, we cultured SY14 and BY4742 in YPD (glucose) media +/− 5% ethanol (Fig. 2c). The lag phase after inoculation was longer for SY14 with 5% ethanol (Fig. 2d), and the doubling time during growth on glucose increased by 55% for BY4742 and 100% for SY14 (Fig. 2e). These findings suggest that SY14 is sensitive to ethanol, even in the presence of glucose. This sensitivity may influence the observed increase in cell death in the SY14 background (Fig. 2f).

### SY14 exhibits decreased expression of diauxic shift related genes in the ethanol phase

**Fig. 3:**
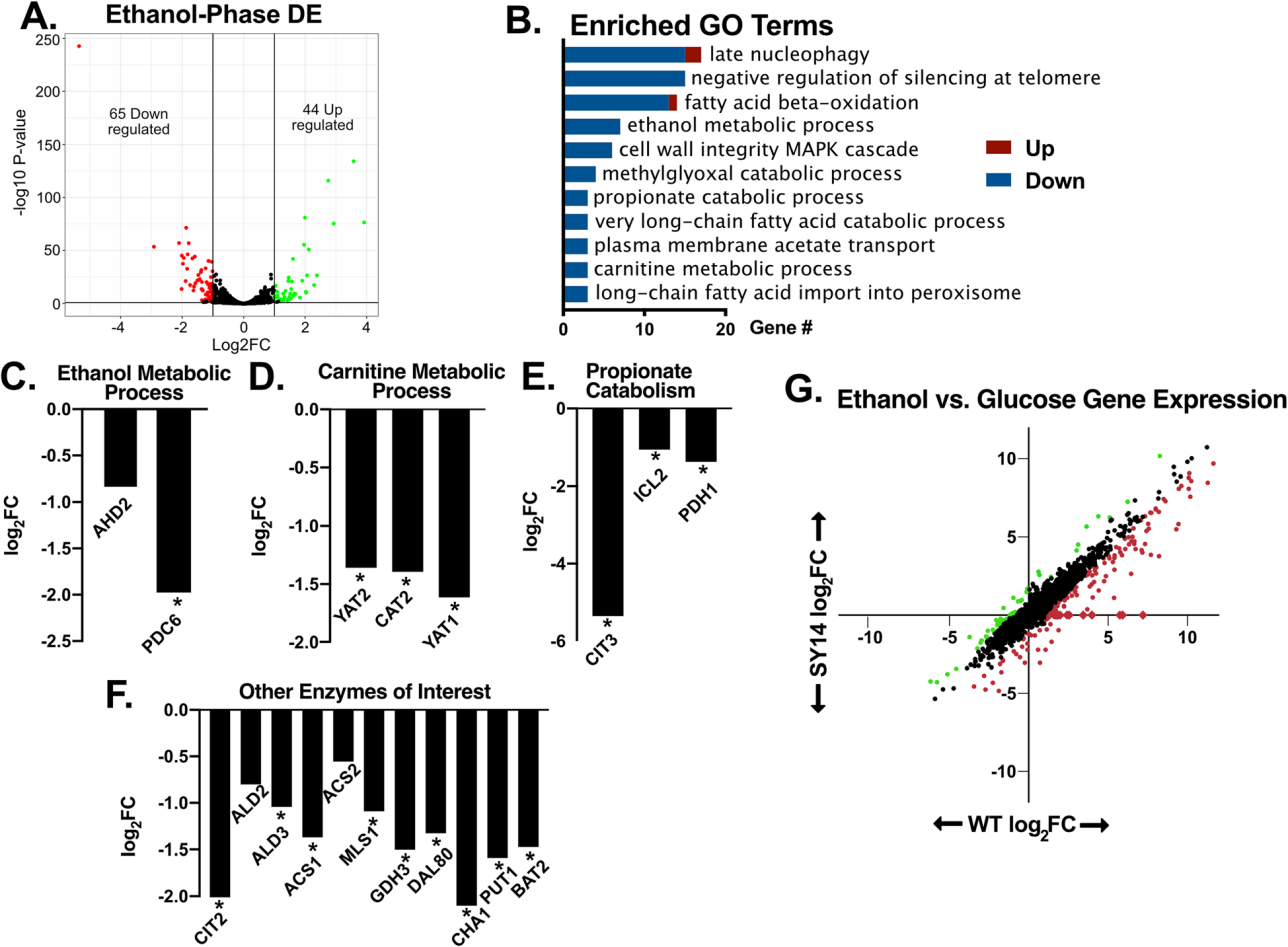
Ethanol-phase RNAseq shows decreased induction of diauxic shift genes in the single chromosome strain. **a** A volcano plot showing the differentially expressed genes in SY14 versus control after 20 h of growth. **b** Downregulated genes from ethanol-phase RNAseq were assessed for enriched GO terms for strain SY14. **c**-**f** Individual differential expression results are shown for select GO terms and genes. * indicates log_2_FC < − 1 FDR < 0.01. **g** Gene expression changes between glucose and ethanol phase cultures of WT and SY14 are shown for comparison. Genes that exhibited increased (green) or decreased (red circles) expression in SY14 compared to the reference are highlighted

Analysis of transcriptomic measurements during growth on glucose discovered relatively few differentially expressed genes in the SY14 background (53 genes) (Additional File 1: Fig. S4a, Additional File 2). The number of differentially expressed genes is intriguing as chromosomal fusion drastically altered genome arrangement and disrupted many interchromosomal interactions, which are important for gene regulation in higher eukaryotes [17–19]. Furthermore, chromosomal fusion removed the majority of telomeres and centromeres which have previously been shown to influence gene silencing [13, 20]. However, these gene expression results may not encapsulate the deficiencies of the strain during growth on non-fermentable carbon sources or in the presence of ethanol (Fig. 2). To further understand these phenotypes, we performed RNAseq to compare gene expression between the reference (BY4742) and single chromosome (SY14) strains during growth on ethanol following a glucose batch phase and the diauxic shift (Fig. 3a). This analysis resulted in identification of a modest number of differentially expressed genes (109). Interestingly, genes with significantly lower expression in SY14 were enriched for functions related to growth on non-fermentable carbon sources (Fig. 3b). Specifically, SY14 exhibited lower gene expression for enzymes involved in ethanol, carnitine, propionate, and fatty acid metabolism (Fig. 3c, d, e, f), all of which enable *S. cerevisiae* to generate ATP after glucose depletion. The diminished gene expression observed might predict diminished growth post diauxic shift, which was observed in Fig. 2b.

The diminished activation of the aforementioned genes was not associated with changes in sequence of the ORF, promoters, or terminators, with one exception, *CIT3,* which encodes a mitochondrial citrate and methylcitrate synthase. The promoter and 5′ coding region of *CIT3*, a gene known to be involved in propionate metabolism [21], was removed during construction of the SY14 strain. Reintroduction of *CIT3* via a *URA3*-marked 2 μm plasmid did not alter the growth or ethanol tolerance of SY14 (Additional File 1: Fig. S5), which might be expected as this gene was shown to encode a minor isoform of citrate synthase [21]. Further analysis of the RNAseq data identified several subtelomeric genes that were not expressed in the single chromosome strain (e.g. *HSP33*, *PAU4*, and *AAD4*), analysis of the SY14 genome sequence showed that these genes were likely removed during chromosomal fusion (Additional File 1: Fig. S6). The majority of these deleted genes lacked a functional description (54%), or were members of the duplicated gene families PAU, COS, and AAD (24%). These genes were not amongst gene sets known to be essential [22], associated with slow growth [22], or known transcription factors [23]. Further, as a group, these deleted genes represented a small percentage of the total RNAseq reads in the wildtype strain during glucose (0.10%) or ethanol (0.16%) phase.

Next, we compared gene expression between glucose phase and ethanol phase for each strain to understand the transition between growth on different carbon sources. This analysis showed that some genes that were proximal to the remaining telomeres were upregulated during ethanol phase in the reference strain, but were not upregulated in the single chromosome strain (Additional File 1: Fig. S7), suggesting that SY14 has increased subtelomeric silencing. A previous work described increased Sir3 dosage as a cause for increased subtelomeric localization and repression [24]. This Sir3 dosage mechanism could explain the repression of subtelomeric genes in SY14, as the number of subtelomeric loci is reduced by 16-fold and *SIR3* expression levels were unchanged. Further analysis showed that several metabolic genes that were not telomere proximal exhibited lower induction during ethanol phase in SY14 compared to wildtype strains. We refer to these genes as poorly induced, as they are significantly upregulated (log_2_FC > 1 FDR < 0.01) in the wildtype strain upon transition from glucose to ethanol phase, but were at least two-fold less induced in the SY14 background compared to the reference strain (Fig. 3g red dots). The 111 genes that were poorly induced in SY14 represented 3.56% of all RNAseq reads in ethanol phase samples, in contrast, these genes accounted for 7.1% of reads amongst reference samples. These data suggest that the significantly decreased ethanol phase expression of metabolic genes like *ACS1*, *YAT1*, *YAT2*, *CIT2*, *PDC6*, and *ADH2* in Fig. 3c, d, e, f was due to a failure to upregulate these genes after the transition from glucose to non-fermentable carbon source growth in the SY14 background. The similarity of the functional annotations of the poorly induced genes in SY14 may indicate disruption of a global mechanism for regulating non-fermentable carbon source gene expression.

### Metabolic modeling of SY14 predicts an ATP bottleneck during ethanol growth

**Fig. 4:**
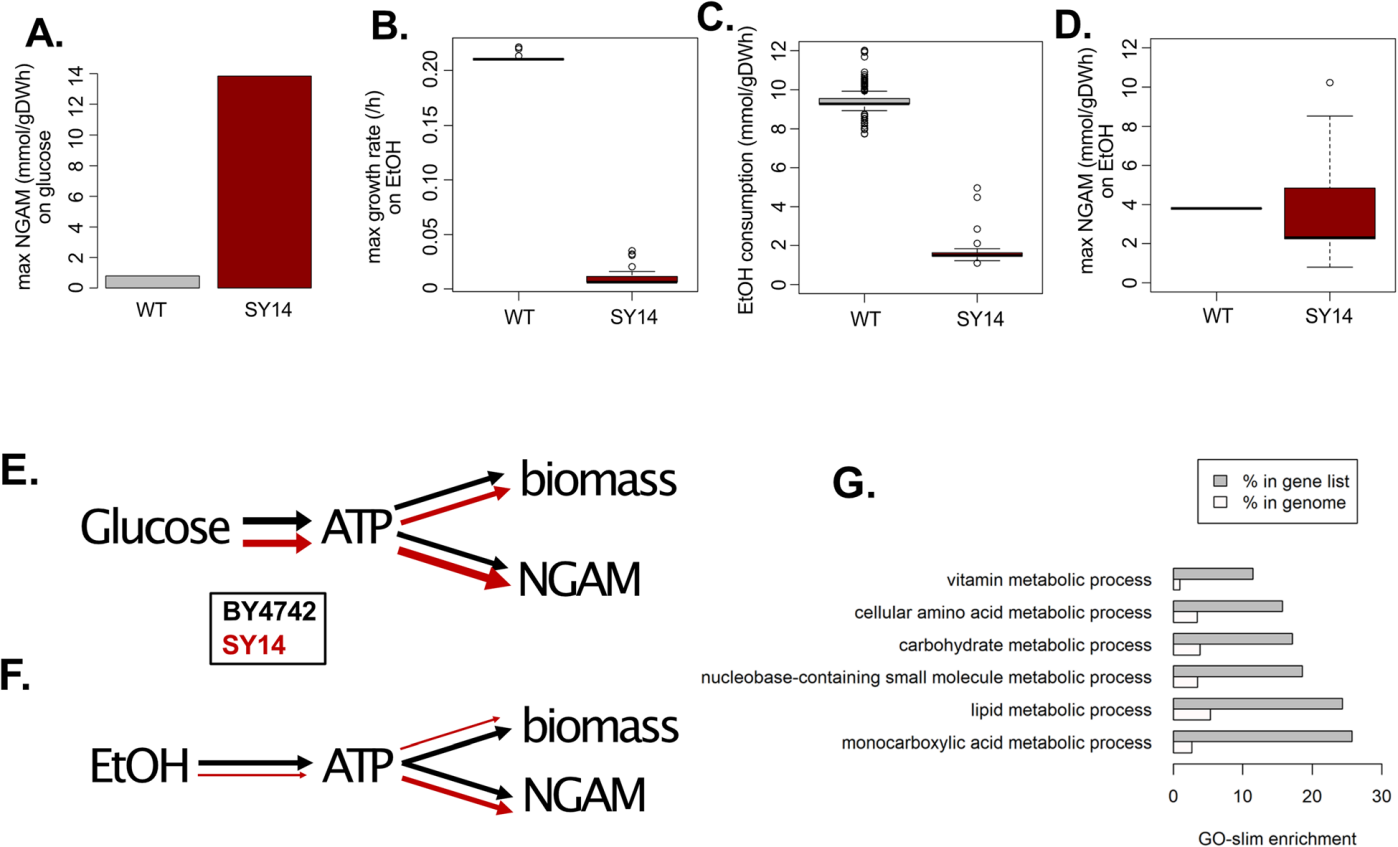
Enzyme-constrained Genome Scale Modeling (ecGEM) predicts high NGAM on glucose and poor ATP generation on ethanol for the single chromosome strain. **a** An ecGEM was constructed to predict wildtype and SY14 ATP expenditure on non-growth associated maintenance (NGAM) when using glucose as the sole carbon source. A model reflecting the disrupted ORFs and differentially expressed genes was constructed to model growth on EtOH as the sole carbon source (**b**), ethanol consumption rates in SY14 (**c**), and max NGAM (**d**). Diagrams representing the growth defects in SY14 when using glucose (**e**) or EtOH (**f**) as the sole carbon source. Line thickness is indicative of relative flux for reference (black) and SY14 (red). **g** Enzymes predicted to rescue growth defect of SY14 on ethanol were enriched for GO terms related to energy generation

Our transcriptomic and genomic analyses showed that 248 enzyme-encoding genes exhibited altered expression (FDR < 0.05 compared to reference) in the SY14 background during growth on ethanol. These genes and/or the subtelomeric genes that were disrupted during chromosomal fusion (Additional File 1: Fig. 6), might explain the growth phenotype of the SY14 strain. To further understand how these changes in expression and deletions might influence glucose and non-fermentable carbon source growth, we constructed enzyme-constrained Genome-scale Metabolic Models (ecGEM) for both reference and SY14 strains [25], using the magnitude of gene expression changes to constrain enzyme usage in SY14 relative to the reference. As SY14 and reference cells exhibited remarkably similar metabolic profiles (Fig. 1) and gene expression profiles (Additional File 1: Fig. S4) in glucose-phase growth, the minor growth defect of SY14 pointed to an increased ATP expenditure for non-growth associated maintenance (NGAM), indicating that resources were being diverted to deal with stress (Fig. 4a).

In contrast, modeling growth on ethanol as the carbon source showed that differentially expressed metabolic enzymes drastically limited the ability of SY14 to grow (Fig. 4b) and utilize ethanol (Fig. 4c). Of note, the calculated maximum ATP expenditure on NGAM was comparable between wildtype and SY14 during growth on ethanol (Fig. 4d), indicating that a bottleneck in ATP generation from ethanol underlies the reduction in biomass formation for SY14. Together, this analysis suggested that when using glucose as a carbon source, the growth defect in SY14 cells arose from an increased ATP expenditure to handle stress (Fig. 4e). Conversely, the model predicts that reduced cell growth on ethanol was a result of a disruption in metabolism leading to a reduced capacity to generate energy (Fig. 4f). In silico rescue experiments identified 70 of the 248 perturbed enzyme-encoding genes as candidates that could rescue the ethanol-phase growth defect of SY14 (Additional File 3). Some of these genes (7/70) were deleted during chromosome fusion and were members of multicopy gene families whose individual contributions to metabolism are unclear. The remaining genes (63/70) were downregulated metabolic enzyme-encoding genes whose coding sequences were not perturbed in SY14, like *ACS1*, *PDH1*, and *YAT1*. The 70 rescue candidate genes were enriched amongst GO-slim terms related energy generation, including lipid metabolism, nucleotide metabolism, and carbohydrate metabolism (Fig. 4g), consistent with our model of SY14 showing a growth defect using ethanol as the carbon source in Fig. 4f.

### SY14 growth on ethanol is rescued by the sirtuin-family deacetylase inhibitor nicotinamide

**Fig. 5:**
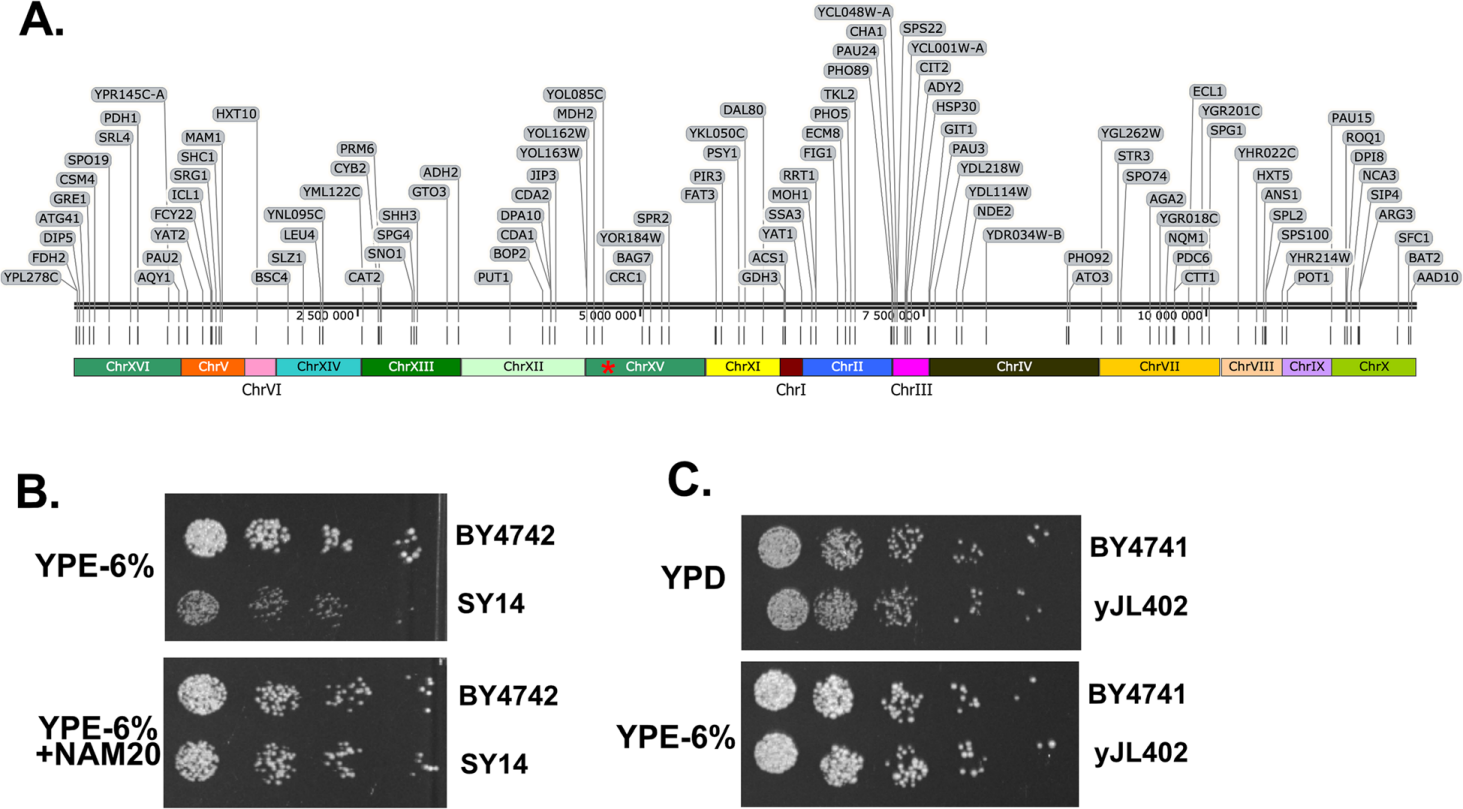
Diminished ethanol growth may be due to widespread sirtuin-mediated silencing in SY14. **a** The genome of the single chromosome strain (SY14) is shown with poorly induced gene names and locations in gray. A red * marks the location of the remaining centromere. **b** Serial dilutions of BY4742 (reference) and SY14 are shown in the presence or absence of 20 mM nicotinamide (NAM). **c** A reference (BY4741) and a two chromosome strain (yJL402) were grown on glucose or ethanol as the sole carbon source

Figure 3 shows diminished induction of several genes involved in metabolic processes that are expected to be upregulated after the diauxic shift. These poorly induced genes were distributed throughout the genome in the single chromosome strain (Fig. 5a), which suggested that the change in expression was not restricted to a single locus. One possibility is that these widespread changes could occur due to a disturbance in chromatin silencing complex activity. During SY14 construction, ~ 300 kb of subtelomeric DNA was removed to facilitate chromosomal end-to-end fusions (Additional File 1: Fig. S6b). These subtelomeric regions represent ~ 2.6% of the genome, but in part due to sirtuin family histone deacetylase mediated silencing [26], these regions represent only 0.1–0.2% of total transcripts in our reference data (Additional File 1: Fig. S6b, S6c). Further, sirtuins have been shown to downregulate widespread genes when subtelomeric structures are disrupted in *S. cerevisiae* [20]. To test whether diminished ethanol growth for SY14 was influenced by sirtuins, growth assays were performed in the presence of nicotinamide (NAM), an inhibitor of sirtuin family deacetylases [26]. Growth on ethanol for SY14 was rescued by NAM (Fig. 5b), indicating that the enzymes that are necessary to efficiently catabolize ethanol are encoded in the SY14 genome, but that these genes, or their activators, may be repressed by sirtuins. This finding is congruent with the poor induction of genes like *CIT2*, *ACS1*, and *PDC6* in the ethanol phase for SY14 compared to the reference strain (Fig. 3).

The above findings suggest that sirtuin-mediated silencing impedes growth on ethanol in the SY14 background. Next, the ethanol sensitivity of an independently constructed chromosomal fusion strain that has two large chromosomes (yJL402) was assessed [14]. Unlike SY14, the yJL402 growth was similar to the reference strain with ethanol as the sole carbon source (Fig. 5b). These data show that *S. cerevisiae* can thrive on ethanol with major changes to chromosome size, subtelomeric DNA content, and a large number of deleted subtelomeric ORFs. Specifically, yJL402 harbors two very large chromosomes (6 Mb each) instead of 16, lacks 200 kb of subtelomeric DNA, and 70 subtelomeric ORFs compared to the reference strain. In contrast, SY14 harbors a single chromosome (12 Mb), lacks 300 kb of subtelomeric DNA, and 107 subtelomeric ORFs (Additional File 1: Fig. S6b). Fifty-seven ORFs are absent in both SY14 and yJL402, including multiple members of subtelomeric gene families, such as Aryl Alcohol Dehydrogenases (AAD), Conserved Sequence (COS) genes, seripauperin (PAU) genes, and YRF family helicases (Additional File 4). In each case, SY14 lacks more members of each of these families compared to yJL402. Another possibility is that one or more of the 51 ORF deletions unique to SY14 cause diminished ethanol growth. Of these genes, only *THI12, YEL073C, AAD14, THI13, YGL258W-A, SNZ2, YGL262W*, and *COS6* were expressed at > 1 mRNA copy per cell (> 25 transcripts per million) during the ethanol phase in our reference dataset (Additional File 4).

## Discussion

In this work we investigated an *S. cerevisiae* strain whose genome is packaged into a single chromosome (strain SY14). Our interest in this strain stemmed from previous observations that a vast increase in chromosome length, a 2.6% reduction in genome size, and 107 deleted genes in relation to the reference strain resulted in only subtle changes in growth [13]. Herein, we identify a more striking phenotype for strain SY14: diminished growth on ethanol. We observed that downregulated genes for SY14 compared to the reference strain during growth on ethanol were significantly enriched for non-fermentable carbon source GO terms. This was surprising as the majority of these genes had unaltered coding sequences, promoters, terminators, as well as upstream and downstream genes in SY14 compared to the reference strain. Metabolic models that simulated decreased flux through the enzymes that correspond to the under-induced genes (e.g. Ald3, Acs1, and Pdh1) predicted diminished ATP generation for SY14 on ethanol, which may explain diminished biomass accumulation. Exometabolite analysis of fermentations suggested a bottleneck to ethanol catabolism downstream of alcohol dehydrogenase, which could result in an accumulation of intermediates of ethanol catabolism in SY14, some of which (e.g. acetaldehyde) are toxic [27] and could cause the observed ethanol sensitivity. These observations provide a plausible cause/effect relationship between decreased expression of key ethanol growth genes and diminished growth on ethanol in SY14.

The observations above suggest that a genomic change in SY14 disrupts gene induction after the diauxic shift. We hypothesized that poor induction could be caused by repression and observed that SY14 was rescued by the addition of nicotinamide (NAM), an inhibitor of sirtuin-mediated gene silencing activity. This rescue result indicates that the genes necessary for ethanol growth and metabolism are present in SY14, but that one or more of these is repressed. Previous works suggest that sirtuins connect the metabolic state of yeast to gene expression responses by sensing NAD+ [28, 29], and deletion of *SIR2* has been shown to enhance ethanol catabolism [30] and gluconeogenesis [31]. Further, the majority of genes that are upregulated in sirtuin deletion strains are also significantly upregulated in our reference strain during growth on ethanol (Additional File 1: Fig. S8) [28, 32]. These repressed genes include genes encoding enzymes involved in non-fermentable carbon source growth including *CIT2*, *PDC6, YAT1,* and transcription factors including *ADR1*, which influences post-diauxic gene expression [28, 32]. These data suggest that the switch-like change in gene expression upon glucose depletion in *S. cerevisiae* may be driven by alleviation of sirtuin-mediated silencing of specific genes. If this is the case, then the diminished growth of SY14 could be the result of incomplete de-repression of post-diauxic growth genes in the absence of glucose. Conversely, a past report identified Sir2, Hst1, and Sum1 as repressors of genes involved in glycolysis during the diauxic shift [33]. Together these data suggest a mechanism driven by NAD+ and Sir2 family deacetylases that matches gene expression regimes to carbon source availability. Additional analyses of the single chromosome strain with Sir2 family deacetylase deletions and/or chromatin IP of Sir3 or Sum1 are needed to thoroughly test this hypothesis.

While the addition of NAM rescued growth on ethanol, it remains unclear which genomic alterations cause the SY14 ethanol growth phenotype. To investigate this, we analyzed a two chromosome strain (yJL402) described in Luo et al. 2018 [14]. Despite vast increases in chromosome size, the removal of 28 telomeres and 200 kb of subtelomeric DNA, and 70 deleted genes, yJL402 did not exhibit diminished growth on ethanol. The NAM rescue and two chromosome growth findings could indicate that one or more of the deleted or repressed genes unique to SY14 (in relation to yJL402) is required for transcriptional reprogramming of ethanol growth genes. We note that *HSP33* and *HSP34* have been linked to the reprogramming of gene expression during the diauxic shift, and that these genes were deleted in SY14 and yJL402 [34]. However, it is possible that these genes, along with other gene deletions or repressions unique to SY14 could cause diminished growth on ethanol. For example, *HSP32* may be repressed by the remaining telomere in SY14 at a key point during the fermentation (e.g. during the diauxic shift), leading to an additive effect to the deletion of *HSP33* and *HSP34.* However, we note that *HSP32* expression was below the limit of quantification for wildtype and SY14 at the timepoints we measured in this work. Another possibility is that the genomic re-organization that has been observed upon glucose exhaustion [35–37] is disturbed in SY14, either due to the presence of only two telomeres or the single very long fusion chromosome. This possibility is supported by the altered 3D organization of the genome of SY14 compared to reference during glucose growth [13], but has not been tested during growth on ethanol. Finally, the observations might suggest that the removal of subtelomeres, which are repressed by sirtuins [26], results in repression elsewhere. Notably, 14 members of the PAU family of genes are deleted in SY14 versus seven in yJL402, and PAU genes have been identified as recruitment sites that facilitate sirtuin-mediated silencing [38]. Further, disruption of sirtuin recruitment to telomeres has been shown to lead to repression of genes throughout the genome [20].

## Conclusions

Modern synthetic biology techniques have enabled the construction of strains with vast genomic alterations, such as the single chromosome yeast (SY14). This strain lacks 30 subtelomeres and 300 kb of subtelomeric DNA, which was removed as 16 chromosomes were fused into one large chromosome [13]. Herein, we found that the SY14 strain exhibited significantly depleted induction of genes involved in non-fermentable carbon source utilization, which metabolic models predicted would cause significantly diminished ATP production in the SY14 strain during growth on ethanol. Indeed, SY14 exhibited severely diminished growth with ethanol as the sole carbon source, which was rescued by nicotinamide, an inhibitor of sirtuin-family deacetylases. This work suggests that sirtuin-mediated transcriptional tuning facilitates ethanol metabolism via transcriptional derepression of non-fermentable carbon source genes. These findings suggest that SY14, a strain that lacks many sirtuin binding and silencing sites (i.e. 30 subtelomeres), is a promising experimental system for future research in the field of sirtuin-mediated gene regulation.

## Methods

### Strains and cultivation conditions

The wildtype (BY4742) and single chromosome strain (SY14) were acquired from the lab of Zhongjun Qin and were grown at 30 °C throughout this work. The independently constructed two-chromosome strain yJL402, and its wildtype control BY4741, were acquired from the lab of Jef Boeke. The batch fermentations in Fig. 1 were carried out in YPD media with 1% glucose in a 500 mL working volume bioreactor. Strains in Fig. 2 and in Additional File 1 were cultivated in YPD with 2% glucose (liquid media), or YP agar with 2% glucose, 3% glycerol, 3% ethanol or 6% ethanol inoculated from YPD pre-cultures grown for 48 h. For Fig. 5, strains were cultivated on YP agar with 2% glucose, 6% ethanol, or 6% ethanol and 20 mM nicotinamide (Sigma N0636-100G). For Additional File 1: Fig. S5, the *CIT3* ORF was expressed from a *Kl*URA3 marked 2 μm plasmid flanked by the endogenous *CIT3* promoter and terminator. A control plasmid was constructed from the aforementioned construct by removing the *CIT3* ORF. Strains expressing the control and *CIT3* plasmids were cultivated in synthetic complete media lacking uracil.

### Analysis of doubling time, lag phase, and final OD

Doubling times were calculated using a non-linear fit of the exponential phase of glucose growth for each strain. This analysis was based on CO_2_ evolution for Fig. 1 and OD measurements for Fig. 2, and Additional File 1:Fig. S2. Lag-phase measurements were defined as the time elapsed for the first 1.5 doublings, which represents the time between the initial inoculation at 0.1OD_600_ to the cultures reaching 0.25OD_600_. Final OD was measured after 5 days of growth.

### Exometabolite measurements

Extracellular metabolites including glucose, ethanol, glycerol, pyruvate, and acetate, were quantified using an HPLC system (ultimate 3000 HPLC, Thermo Fisher) with a BioRad HPX-87H column (BioRad) and an IR detector, with 5 mM H_2_SO_4_ as the elution buffer at a flow rate of 0.6 mL/min and an oven temperature of 45 °C.

### Collection and analysis of RNAseq data

Biomass for RNAseq was collected in mid-glucose phase (7.5 h after inoculation) and during ethanol phase (20 h after inoculation). RNA extractions were performed on samples that were mechanically lysed with 0.5 mm acid washed beads using an MP-Biomedicals™ FastPrep-24 for three one-minute cycles. Further extraction was performed using an RNeasy® Kit from Qiagen. Libraries were prepared using the TruSeq mRNA Stranded HT kit. Sequencing was carried out using an Illumina NextSeq 500 High Output Kit v2 (75 bases), with a minimum of 8 million paired-end reads per replicate. The Novo Nordisk Foundation Centre for Biosustainability (Technical University of Denmark), performed the RNA sequencing and library preparation. RNAseq was mapped with STAR and reads were assigned with featureCounts. Differential expression results were generated using scripts from the OrthOmics pipeline (https://github.com/SysBioChalmers/OrthOmics) from Doughty et al. 2020 [39], which is based on the limma and edgeR R packages. Dubious ORFs and genes with < 1 Count Per Million (CPM) in three conditions were excluded from differential expression analysis. Raw datasets were uploaded to SRA under the accession number PRJNA594518 and differential expression results are reported in Additional File 2. Gene Ontology analysis was performed with the R-package Piano.

### Metabolic modeling

The genome-scale metabolic model ecYeast 9.3 [40] was used to generate enzyme constrained models for both reference (BY4742) and SY14 strains using the GECKO toolbox [25]. The default enzyme pool parameter of 0.1 was used as the upper limit for enzyme abundance. To model glucose growth, glucose was set as the sole carbon source, and exchange fluxes such as glucose uptake rate, by-product production rates, and biomass formation rate were constrained to observed values for both reference and SY14 strains. The non-growth associated maintenance energy (NGAM) reaction for each model was set as the objective function, and flux balance analysis (FBA) was used to calculate the maximum NGAM for both reference and SY14 strains. To model ethanol growth, we constructed models for both reference and SY14 strains as follows: first, the default ecYeast9.3 model was constrained with ethanol as the sole carbon source and growth rate constrained to 0.22 h^− 1^, with a flexibilization factor of ±5%. We then performed random sampling with a pair of randomly weighted objective functions to obtain a set of 1000 feasible flux distributions in reference strain cell growth [41]. Then, for each flux distribution for the reference strain, we constrained the upper bound of enzyme exchange reactions of the 248 differentially expressed enzymes in SY14 by multiplying the simulated enzyme usage in the reference strain model with the fold-change value in gene expression analysis, with a flexibilization factor of ±20%. The objective function was set first to maximize growth rate; infeasible solutions (4 out of 1000) were discarded. Then, with the growth rate constrained to the maximum calculated value with a flexibilization factor of ±5%, the objective function was set to maximize both ethanol consumption and NGAM. For the in silico rescue experiment, we removed the constraints on the 248 enzymes one at a time for each of the 996 feasible solutions and used FBA to calculate the maximum growth rate using ethanol as the sole carbon source. The average maximum growth rate was calculated, and the 70 enzymes that rescued the mean growth rate to 0.22 h^− 1^ with a flexibilization factor of ±5% are subjected to GO-slim enrichment analysis at Saccharomyces genome database (https://www.yeastgenome.org/). Enriched GO-slim terms with > 5 genes were included.

## Supplementary Information

**Additional file 1: Fig. S1.** Long-term shake flask growth of SY14 results in diminished biomass accumulation. BY4742 and SY14 were grown in YPD media in shake flasks for 10 days. Measurements were taken periodically and are shown in **a**. The difference in biomass on day 10 was 24% (**b**). **Fig. S2.** SY14 does not exhibit increased sensitivity to hydrogen peroxide. **a** Growth curves were calculated using a 48-well growth assay measuring OD_600_ every 10 min for wildtype (BY4742) and single chromosome (SY14) strains. The maximum doubling time during glucose phase (**b**) and lag phase (**c**) are shown. **Fig. S3.** Total protein and rRNA abundance and processing are similar between wildtype and SY14 strains. **a** Protein content was measured via Lowry assay. **b** To assess potential changes in Ribosomal RNA expression or processing, rRNA was measured via qPCR for mature rRNA regions (18S and 25S), as well as a region that is removed during maturation (ITS2). **Fig. S4.** Glucose RNAseq suggests RNR3 and HUG1 are differentially expressed in chromosomal fusion strains grown on glucose. **a** Glucose-phase differential expression data was generated using biomass from fermentations shown in Fig. 1. **b** The data in this report (red) was compared to RNAseq from previous reports that studied either the single chromosome strain (purple) or the two chromosome strain (green). Shared differentially expressed genes (log_2_FC > 1_abs_ FDR < 0.01) were compared amongst up (**b**) and downregulated (**c**) genes. **Fig. S5.** A plasmid borne copy of *CIT3* does not rescue SY14 growth rate or ethanol sensitivity. A plasmid with the *CIT3* promoter and terminator (brown and purple) or plasmid with *CIT3* promoter, ORF, and terminator (dark red and light blue) were used to transform SY14. Growth was monitored in 48-well plate format by measuring OD_600_ every 10 min in the presence or absence of ethanol. **Fig. S6.** SY14 Deleted genes are evolutionarily young genes that are poorly expressed. **a** Chromosome I and II fusion is shown as an example of the cause of ORF removal during the construction of SY14. **b** Genome size and ORF count for wildtype (BY4742) and single chromosome (SY14) strains. **c** The removed ORFs in SY14 were queried for the % of total ORF-associated reads for BY4742 during glucose and ethanol growth. **Fig. S7.** Subtelomeric genes are mis-regulated in SY14. **a** The single chromosome in strain SY14 is shown. The average expression of each of the genes that were detected (> 0.1 Counts Per Million) in at least one sample in our RNAseq datasets are shown for the left telomere (**b**) and the right telomere (**c**). **Fig. S8.** Genes repressed by sirtuins overlap with genes induced during ethanol growth **a** Gene expression during growth on glucose versus ethanol from this report is shown for the 153 genes that were upregulated in a sir2Δhst1Δ strain in Humphrey et al. 2020 [1]. 18 genes from this dataset were not detected in our RNAseq data and were omitted from the analysis. **b** Gene expression during growth on glucose versus ethanol is shown for the 46 genes that were upregulated in a hst3Δhst4Δ strain in Feldman et al. 2019 [2]. For each figure, green indicates a significant upregulation during the ethanol phase, red indicates a significant downregulation, and black indicates no significant change.

**Additional file 2.** Differential expression of SY14 and BY4742 in the glucose or the ethanol phase of growth. RNAseq comparisons that accompany this report.

**Additional file 3.** DE Enzymes Grate. These data accompany Fig. 4 of this work.

**Additional file 4.** Deleted Genes in SY14 and yJL402.

## Data Availability

The RNAseq datasets supporting the conclusions of this article are available in SRA under the accession number PRJNA594518. Differential expression results based on RNAseq data are reported in Additional File 2.

## Abbreviations

AAD: Aryl-alcohol dehydrogenase
COS: Conserved Sequence family of endosomal proteins
CRISPR: Clustered regularly interspaced short palindromic repeats
DNA: Deoxyribonucleic acid
ecGEM: Enzyme-constrained Genome-scale Metabolic Models
GO: Gene ontology
NAD+: Nicotinamide Adenine Dinucleotide
ORF: Open reading frame
PAU: Seripauperin gene family
RNAseq: Assay for quantifying RNA with high throughput sequencing
YPD: Yeast extract peptone dextrose media
