# Supplementary material for: A single chromosome strain of S. cerevisiae exhibits diminished ethanol metabolism and tolerance
Source: BMC Genomics. 2021 Sep 22;22:688. doi: 10.1186/s12864-021-07947-x (PMC8456624; doi:10.1186/s12864-021-07947-x)
Supplement: Supplementary file 1 — Additional file 1: Fig. S1. Long-term shake flask growth of SY14 results in diminished biomass accumulation. BY4742 and SY14 were grown in YPD media in shake flasks for 10 days. Measurements were taken periodically and are shown in a. The difference in biomass on day 10 was 24% (b). Fig. S2. SY14 does not exhibit increased sensitivity to hydrogen peroxide. a Growth curves were calculated using a 48-well growth assay measuring OD600 every 10 min for wildtype (BY4742) and single chromosome (SY14) strains. The maximum doubling time during glucose phase (b) and lag phase (c) are shown. Fig. S3. Total protein and rRNA abundance and processing are similar between wildtype and SY14 strains. a Protein content was measured via Lowry assay. b To assess potential changes in Ribosomal RNA expression or processing, rRNA was measured via qPCR for mature rRNA regions (18S and 25S), as well as a region that is removed during maturation (ITS2). Fig. S4. Glucose RNAseq suggests RNR3 and HUG1 are differentially expressed in chromosomal fusion strains grown on glucose. a Glucose-phase differential expression data was generated using biomass from fermentations shown in Fig. 1. b The data in this report (red) was compared to RNAseq from previous reports that studied either the single chromosome strain (purple) or the two chromosome strain (green). Shared differentially expressed genes (log2FC > 1abs FDR < 0.01) were compared amongst up (b) and downregulated (c) genes. Fig. S5. A plasmid borne copy of CIT3 does not rescue SY14 growth rate or ethanol sensitivity. A plasmid with the CIT3 promoter and terminator (brown and purple) or plasmid with CIT3 promoter, ORF, and terminator (dark red and light blue) were used to transform SY14. Growth was monitored in 48-well plate format by measuring OD600 every 10 min in the presence or absence of ethanol. Fig. S6. SY14 Deleted genes are evolutionarily young genes that are poorly expressed. a Chromosome I and II fusion is shown as an example of the [file 12864_2021_7947_MOESM1_ESM.docx]

**Additional File 1 – Accompanies Doughty et al.**

**Fig. S1**

**
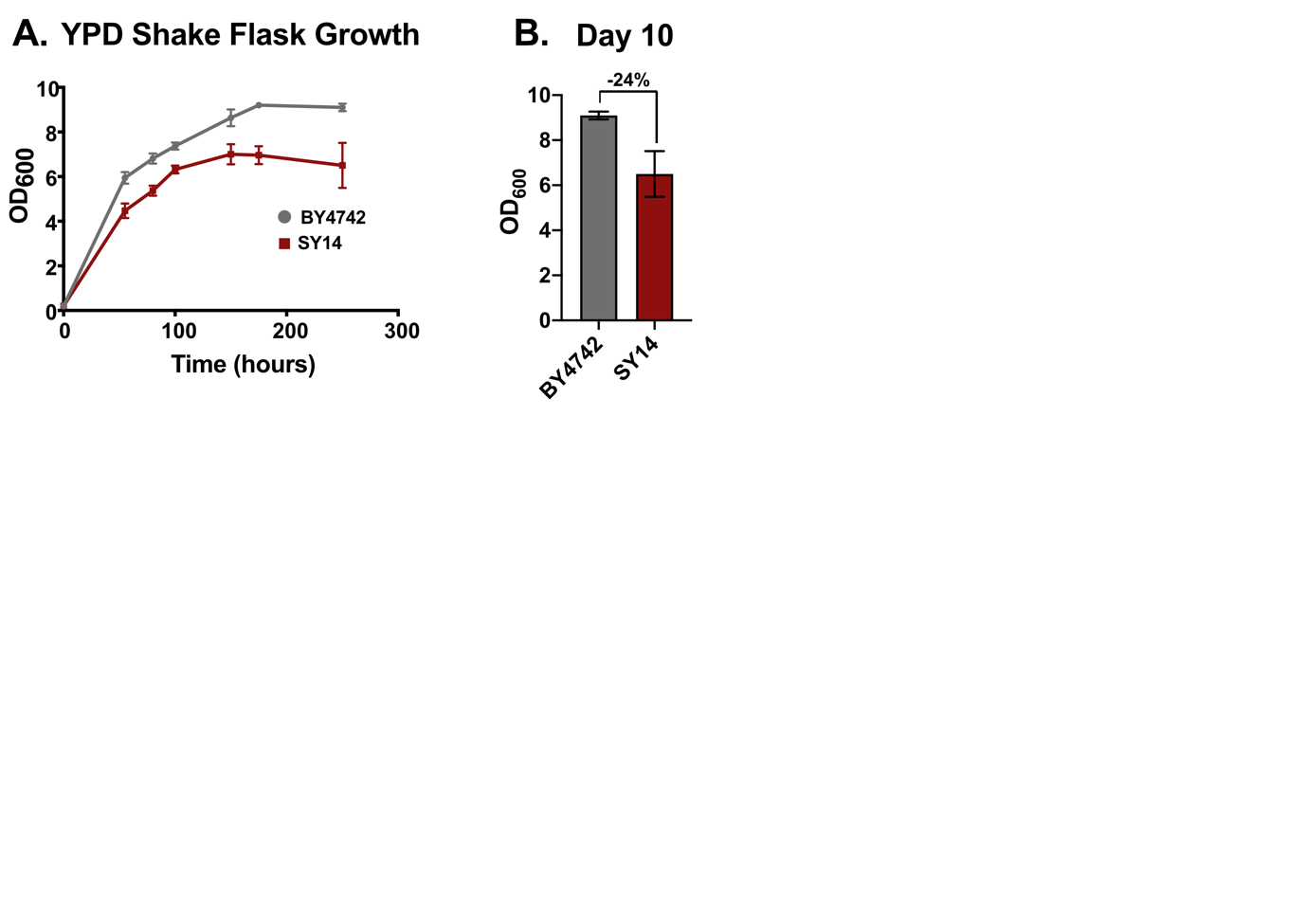
**

**Fig. S2**

**
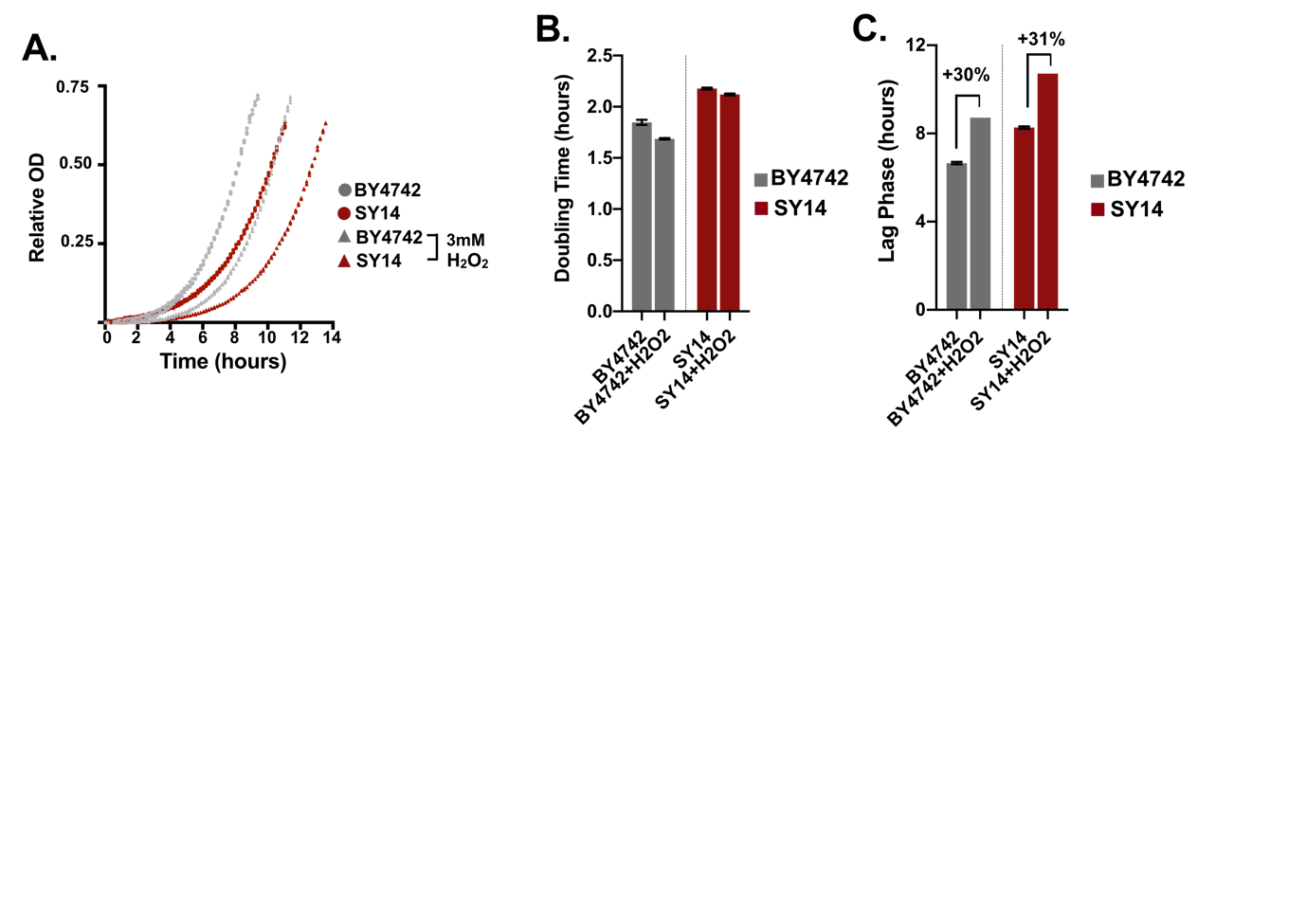
**

**Fig. S3**

**
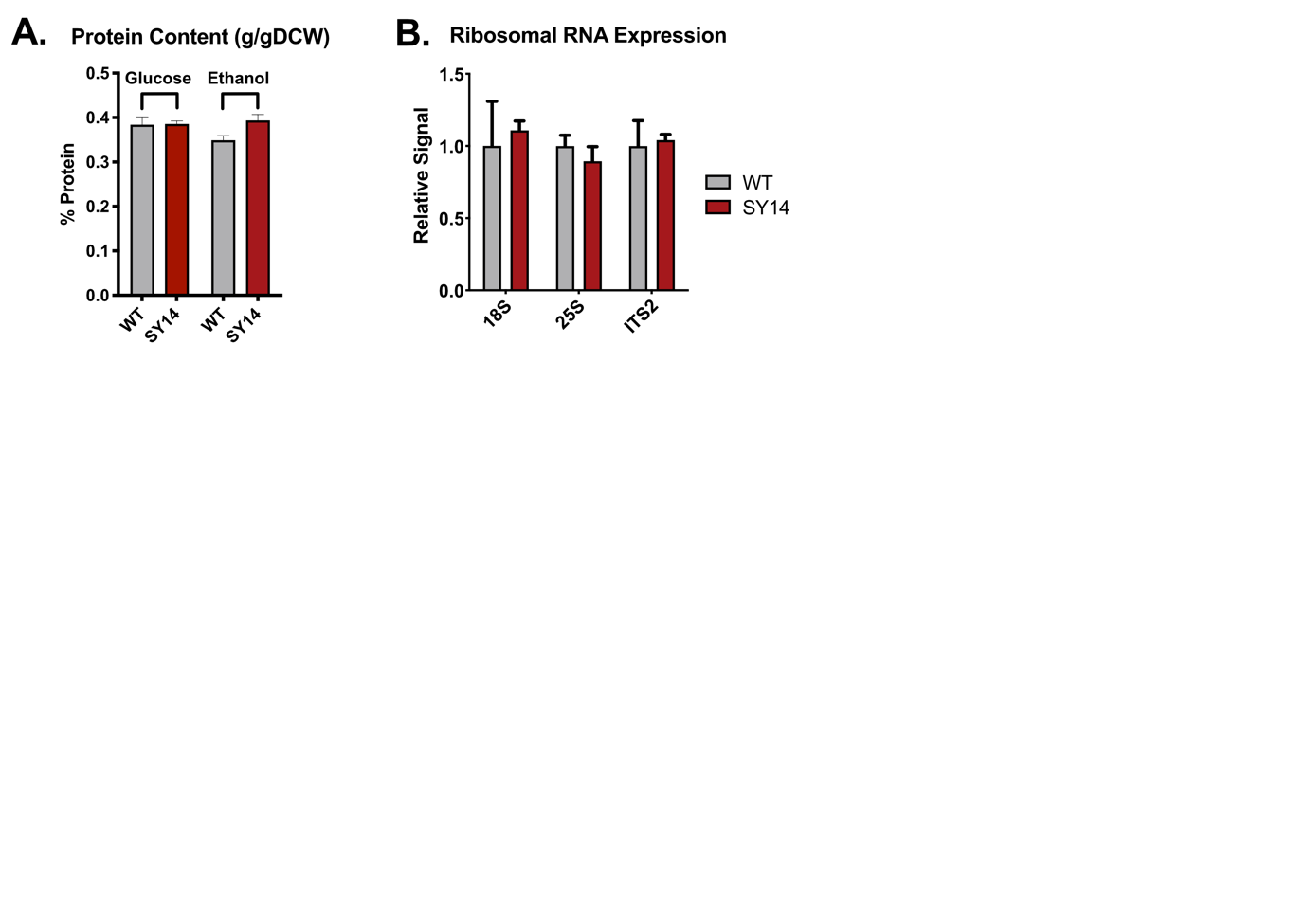
**

**Fig. S4**

**
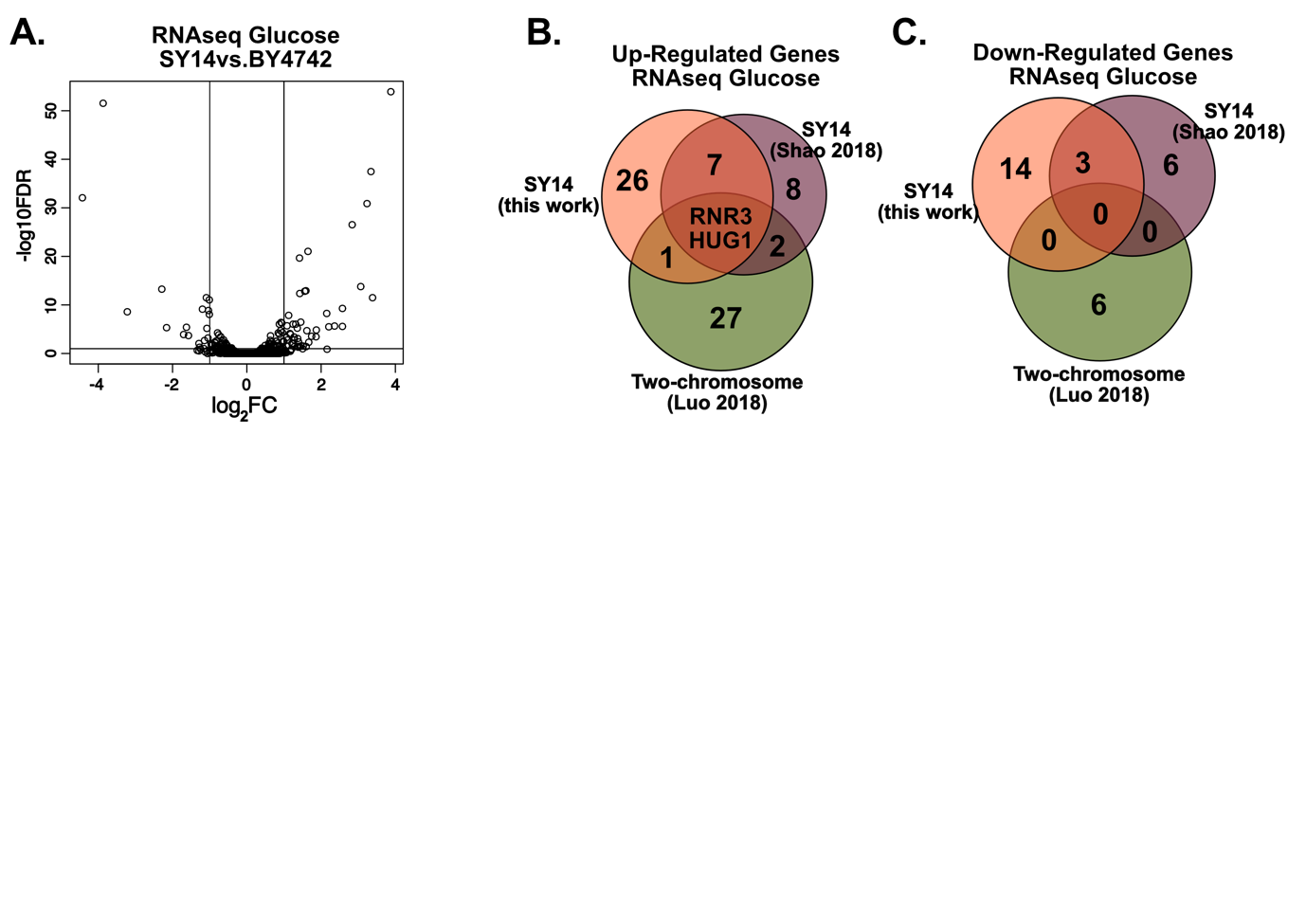
**

**Fig. S5**

**
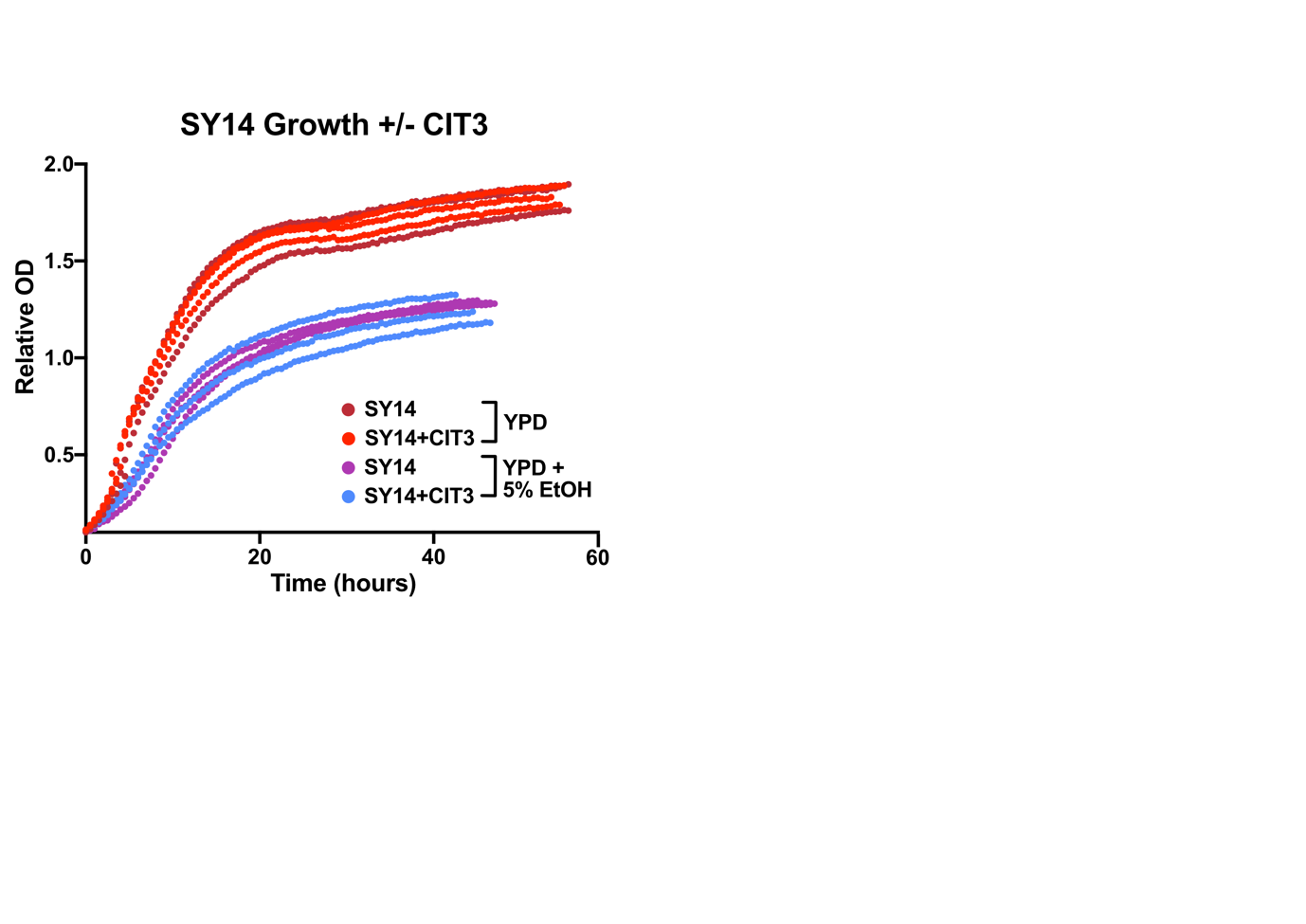
**

**Fig. S6**
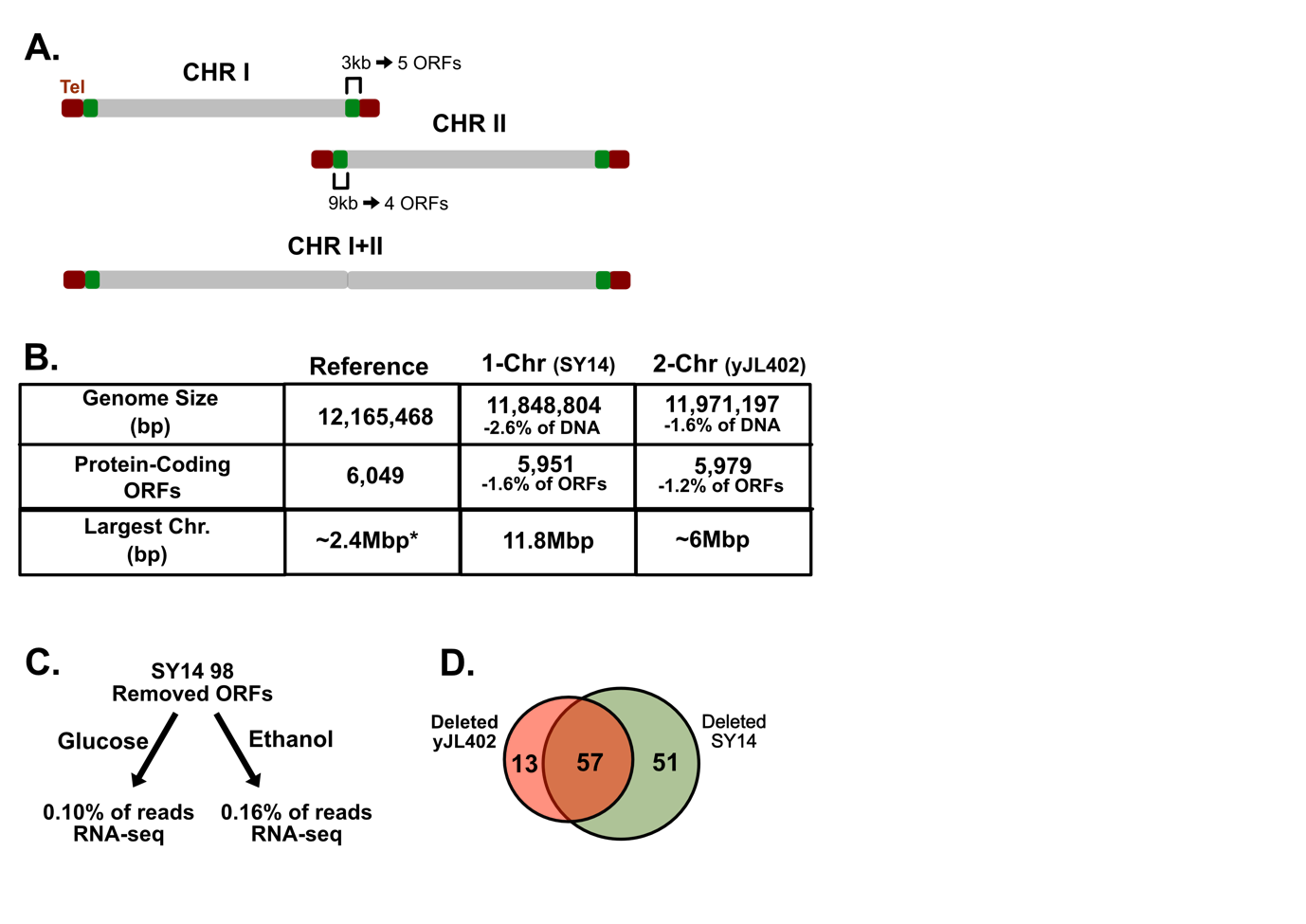


**Fig. S7**

**
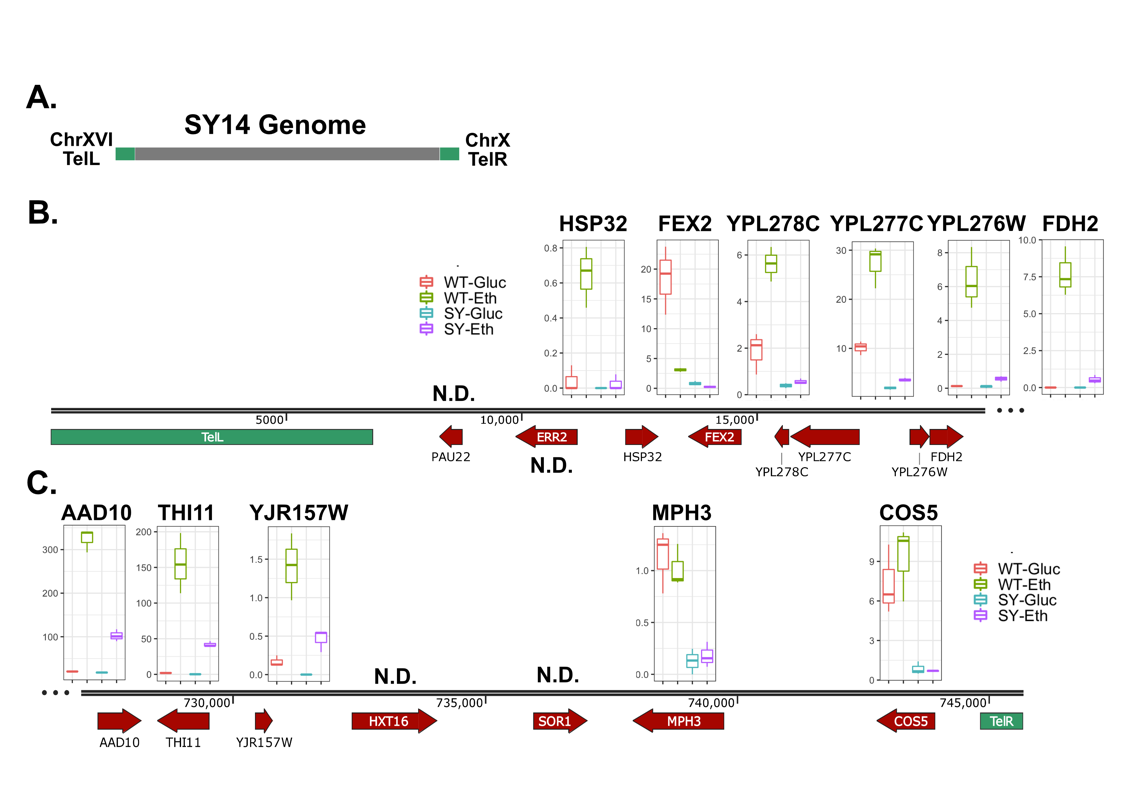
**

**Fig. S8**


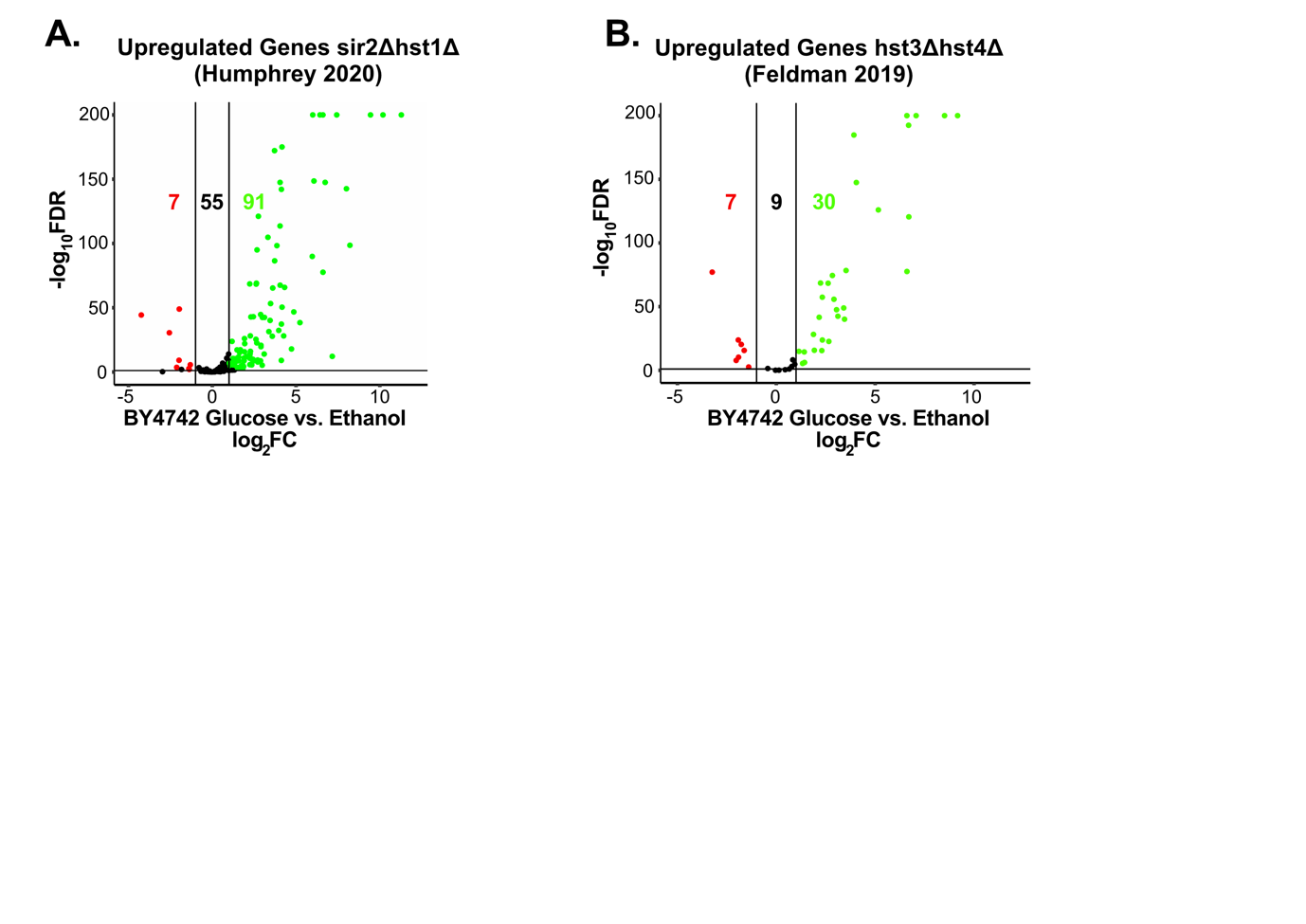


**References**

1. Humphrey, K. M. *et al.* Evolution of Distinct Responses to Low NAD+ Stress by Rewiring the Sir2 Deacetylase Network in Yeasts. *Genetics* 214, 855 LP – 868 (2020).
2. Feldman, J. L. & Peterson, C. L. Yeast Sirtuin Family Members Maintain Transcription Homeostasis to Ensure Genome Stability. *Cell Rep.* 27, 2978-2989.e5 (2019).
